# Supplementary material for: Therapeutic Efficacy of ABN401, a Highly Potent and Selective MET Inhibitor, Based on Diagnostic Biomarker Test in MET-Addicted Cancer
Source: Cancers (Basel). 2020 Jun 15;12(6):1575. doi: 10.3390/cancers12061575 (PMC7352216; doi:10.3390/cancers12061575)
Supplement: Supplementary file 1 [file cancers-12-01575-s001.pdf]

cancers

MDPI

Article

Therapeutic Efficacy of ABN401, a Highly Potent and Selective MET Inhibitor, Based on Diagnostic Biomarker Test in *MET*-Addicted Cancer

Jooseok Kim, Kyung Eui Park, Yoo-Seong Jeong, YeongMun Kim, Hayeon Park, Ji-Hye Nam, Kyungsoo Jung, Woo Sung Son, Hun Soon Jung, Jong-Hwa Lee, Seong Hoon Jeong, Nam Ah Kim, Jae Du Ha, Sung Yun Cho, Yoon-La Choi, Suk-Jae Chung , Jun Young Choi, Sungyoul Hong [1], Young Kee Shin

Supplementary Table

Table S1. Interaction sites between ABN401 and MET protein.

Type of Interaction	ABN401
Hydrophobic Interactions (gray color)	1084I, 1092V, 1108A, 1159Y, 1160M, 1164D, 1167N, 1180A, 1230Y(2), 1231D,
Hydrogen bonds (cyan color)	1084I, 1158P(2) , 1159Y, 1160M, 1161K, 1162H, 1163G, 1167N, 1174H, 1208R, 1209N, 1222D, , 1226A, 1230Y(2)
Pi-stacking interactions (magenta color)	1230Y
Salt bridges	1164D, 1231D(2) , 1233E

(*): numbers of interaction.

Table S2. The summary of kinase selectivity profile at 1 μM ABN401.

Kinase	% Enzyme Activity (Relative to DMSO controls)	
	ABN401	
	Data 1	Data 2
**c-MET**	-0.73	-1.34
**CLK1**	63.51	63.39
**CLK4**	55.62	55.21
**c-MET (P991S)**	37.82	35.90
**c-MET (T992I)**	31.91	29.75
**c-MET (V1092I)**	24.98	24.55
**c-MET (T1173I)**	16.91	16.84
**c-MET (Y1235D)**	6.77	5.63
**c-MET (M1250T)**	45.68	45.09

The kinase selectivity profile of ABN401 was tested in a panel of 571 kinases in single dose duplicate mode at a concentration of 1 μM. The enzyme activity was 0%, 6%, 16.87%, 24.77%, 30.83%, 36.55%, 36.96% 44.59 and 45.39% for c-MET, c-MET(Y1235D), c-MET(V1092I), c-MET(T1173I), c-MET(T992I), CLK1, c-MET(P991S), CLK4 and c-MET(M1250T), respectively.

Table S3. The amount of ABN401 in the tumor tissues at nine time points.

		The amount of ABN401 in the tumor tissues								
**Cell lines**	**Dose (mg/kg)**	**0.5 hr**	**1 hr**	**2 hr**	**4 hr**	**6 hr**	**12 hr**	**24 hr**	**48 hr**	**72 hr**
**EBC-1**	10	436(212)	769(243)	995(82.6)	1260(245)	1020(117)	200(15.9)	54.4(24.1)	13.3(7.39)	N/D
	30	1090(180)	1690(173)	2990(1020)	2950(1010)	2700(505)	732(285)	372(277)	32(2.72)	15.9(3.76)
**SNU-5**	10	387(119)	1230(251)	1390(308)	608(118)	783(38.9)	91.1(12.4)	29.9(12.7)	10.6(2.47)	N/D
	30	731(526)	2450(819)	2920(350)	2230(427)	1780(535)	541(240)	70.9(9.65)	13.5(5.99)	N/D

N/D: Not detectable, (*):± standard deviation.

Table S4. The dose- and time-dependent inhibition of c-MET phosphorylation in tumor tissue.

		**c-MET : phospho-MET ratio _ Immunohistochemistry (IHC)**								
**Cell lines**	**Dose (mg/kg)**	**0.5 hr**	**1 hr**	**2 hr**	**4 hr**	**6 hr**	**12 hr**	**24 hr**	**48 hr**	**72 hr**
**EBC-1**	vehicle	122.32(3.57)	133.04(12.49)	110.41(3.49)	107.93(3.82)	105.79(3.25)	105.64(6.84)	104.04(1.75)	114.95(6.51)	116.82(13.35)
	10	27.77(8.80)	13.69(2.96)	4.647(1.72)	2.53(1.45)	1.88(0.76)	61.07(3.57)	87.39(2.85)	94.45(9.50)	93.62(14.71)
	30	8.92(3.48)	2.84(1.08)	1.45(0.51)	0.45(0.22)	0.47(0.22)	1.14(0.56)	34.89(9.29)	78.29(5.10)	68.54(4.43)
**SNU-5**	vehicle	100.67(0.58)	96.23(4.11)	104.74(6.96)	99.519(3.49)	101.72(3.99)	91.48(4.07)	101.49(4.13)	97.47(2.76)	105.25(9.64)
	10	27.04(4.85)	0.37(0.01)	0.71(0.01)	0.63(0.22)	1.19(0.33)	75.64(5.88)	94.91(3.04)	84.22(4.85)	100.43(6.43)
	30	8.92(3.48)	2.84(1.08)	1.45(0.51)	0.45(0.02)	0.47(0.02)	1.14(0.56)	34.90(9.29)	78.29(5.10)	68.54(4.43)
		**c-MET : phospho-MET ratio _ Westernblotting (WB)**								
**Cell lines**	**Dose (mg/kg)**	**0.5 hr**	**1 hr**	**2 hr**	**4 hr**	**6 hr**	**12 hr**	**24 hr**	**48 hr**	**72 hr**
**EBC-1**	vehicle	67.7(18.63)	57.59(15.72)	37.91(16.35)	55.24(14.06)	27.01(6.66)	93.53(17.93)	97.19(21.75)	60.74(35.02)	41.4(9.68)
	10	7.23(1.69)	5.34(0.27)	7.98(0.63)	4.42(0.11)	3.27(0.55)	50.2(15.94)	157.06(32.28)	137.9(54.82)	118.13(38.55)
	30	8.64(0.77)	7.13(0.75)	10.41(1.00)	4.6(0.27)	3.07(0.76)	41.77(14.84)	164.31(31.52)	102(38.69)	83.76(25.06)
**SNU-5**	vehicle	14.07(0.17)	13.92(0.90)	73.22(25.13)	79.10(40.81)	154.99(25.58)	101.90(21.26)	104.69(45.95)	146.59(29.66)	92.80(17.55)
	10	9.18(2.79)	19.62(7.15)	5.032(0.66)	10.29(1.66)	14.15(2.21)	46.31(12.15)	56.15(19.42)	101.98(18.14)	93.90(9.60)
	30	12.53(2.37)	8.28(0.22)	11.81(1.92)	20.97(2.33)	26.32(3.14)	39.24(7.40)	151.98(16.08)	213.36(17.97)	133.87(14.85)

(*): ± standard deviation.

Table S5. Plasma Protein Binding Assay in mice.

Species	f_{up}
**Mouse**	0.070 ± 0.005

To determine the free fraction of ABN401 in the mouse plasma, rapid equilibrium dialysis (RED) devices (Thermo Fisher Scientific, Waltham, MA, USA) were used in this study. Briefly, an aliquot (200 μL) of mouse plasma containing ABN401 at 1 μM concentration was added to the donor chamber whereas 350 μL of BupH™ phosphate-buffered saline (catalog# 28372) was added to the receiver chamber. The reaction was conducted at 37 °C for 4 hr in a shaking incubator (100 rpm). After the incubation was complete, an aliquot (50 μL) was collected from each side of the chamber. For matrix matching of the samples, a 50 μL aliquot of blank medium was then added to the protein-free solution sample, and an equal volume of protein free solution was also added to the medium. The resulting sample was then analyzed for ABN401 to determine the free fraction in the plasma (f_{up}).

$$f_{up} = \frac{C_{receiver\ chamber}}{C_{donor\ chamber}} \quad (1)$$

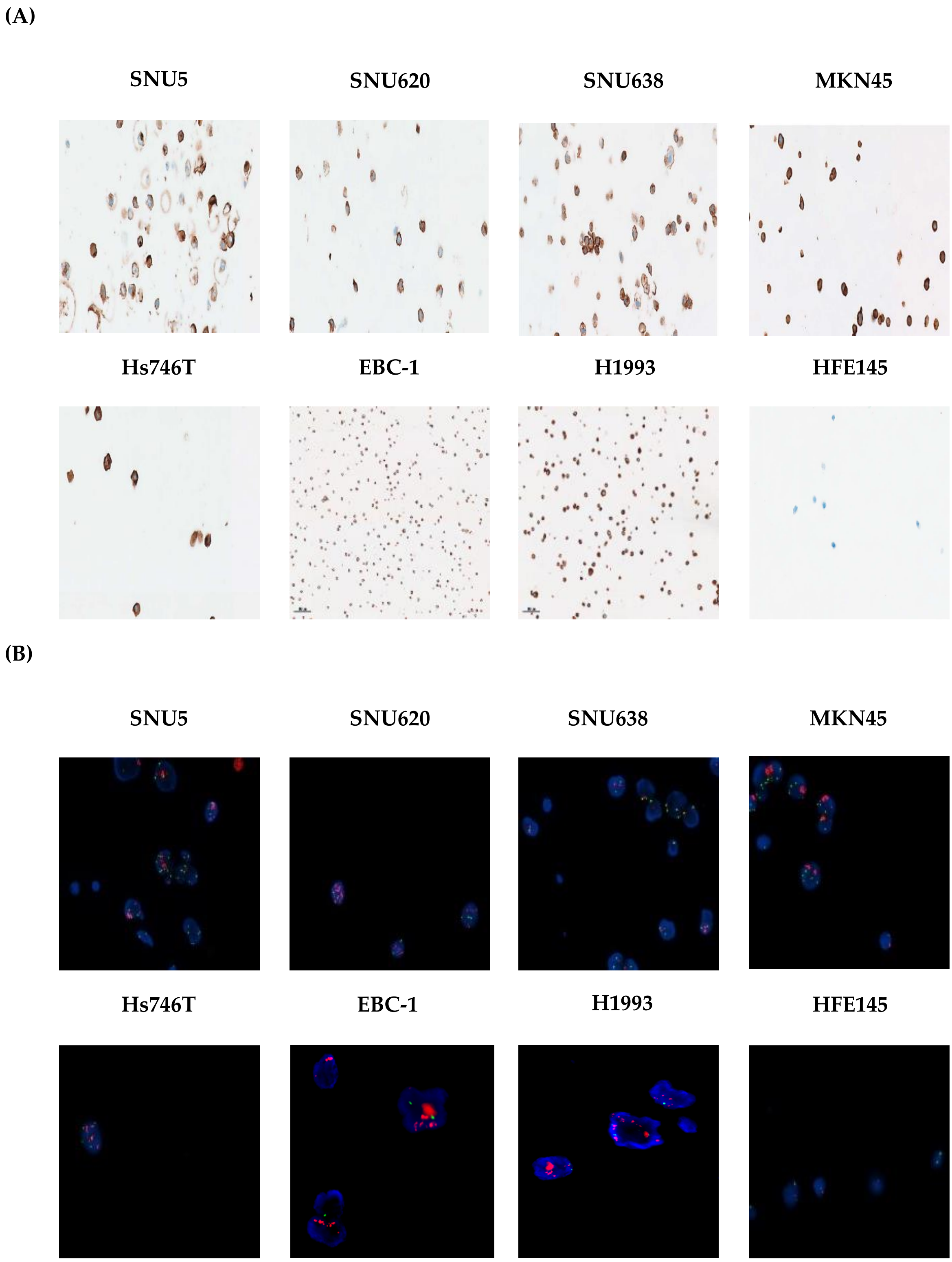

Figure S1. MET status in *MET*-addicted cell lines. (**A**,**B**) c-MET protein expression was measured by immunohistochemistry (IHC), and copy number variation (CNV) was analyzed by fluorescence in situ hybridization in *MET*-addicted cancer cell lines. Except the normal immortalized cell line HFE14 (IHC intensity 0), all cell lines showed IHC intensity 3+ (A). In the FISH analysis, *MET*-high amplified SNU5, SNU620, Hs746T, MKN45, EBC-1 and H1993 cells had a MET/CEP7 ratio >5. SNU638 and HFE145 cells had a MET/CEP7 ratio = 0.8 and 1.0, respectively (B).

* Figure 2B – SNU5

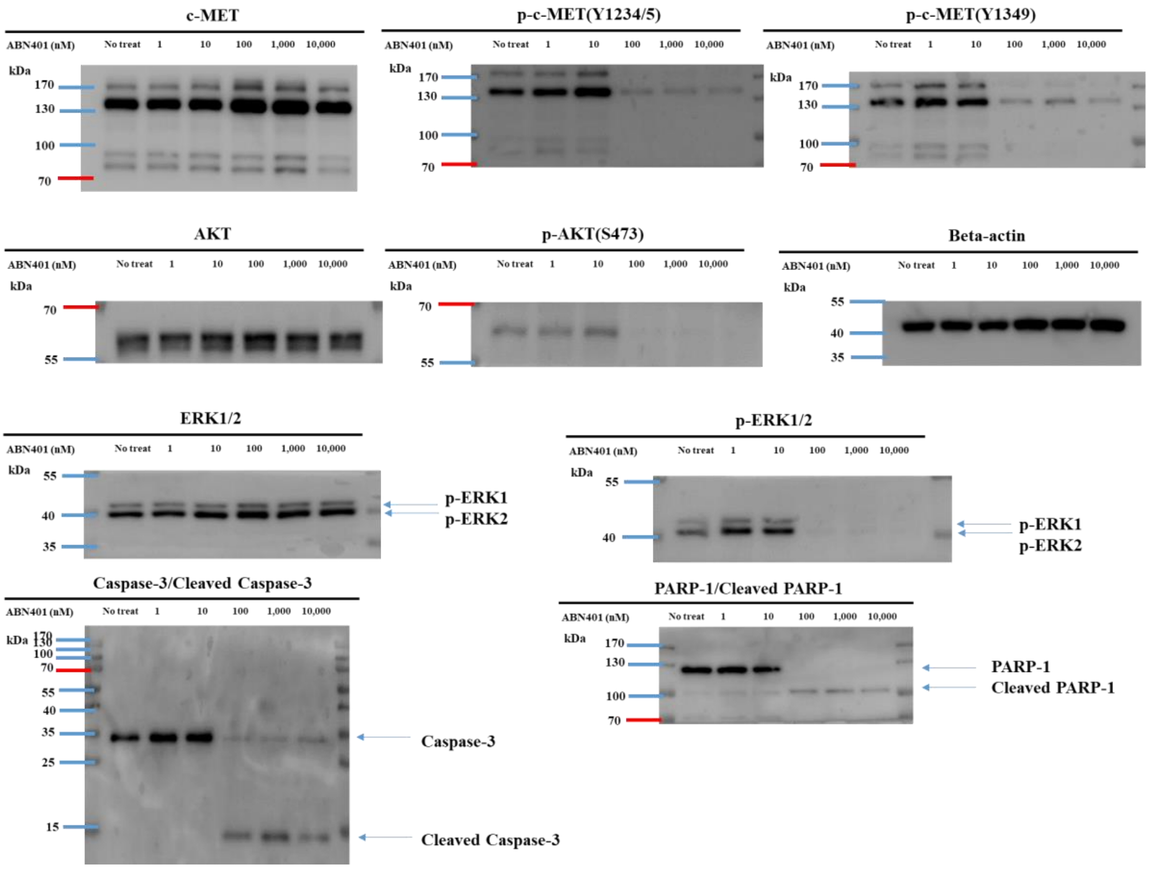

* Figure 2C – Hs746T

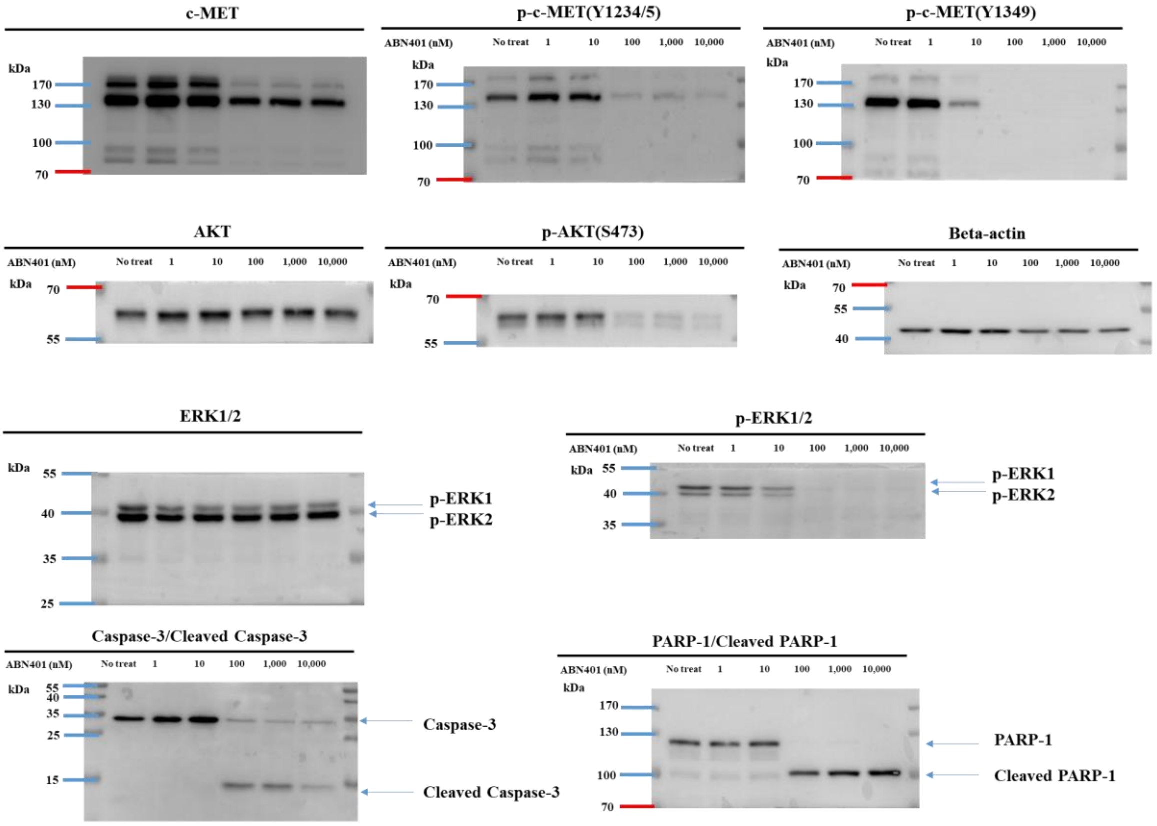

* Figure 2D – EBC-1

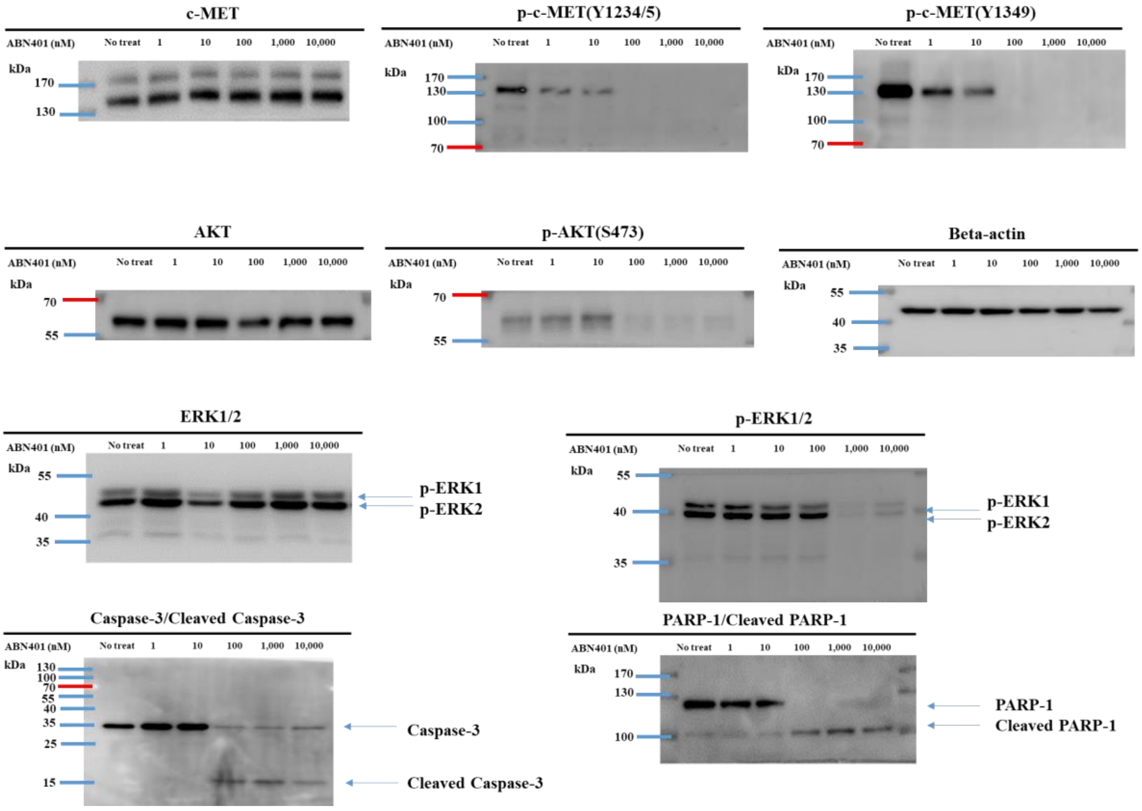

* Figure 2E – SNU638

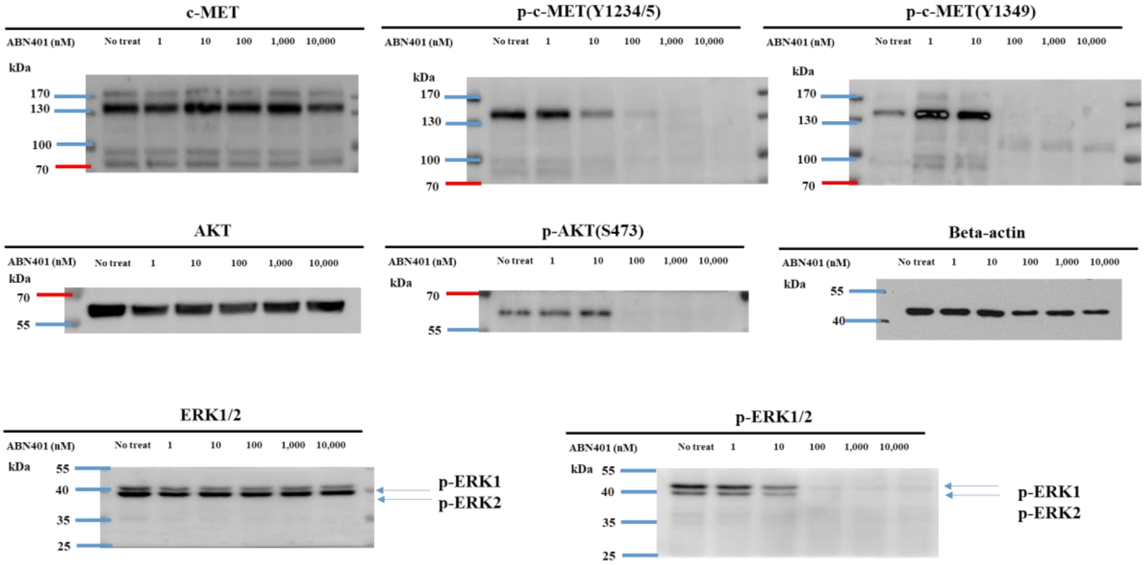

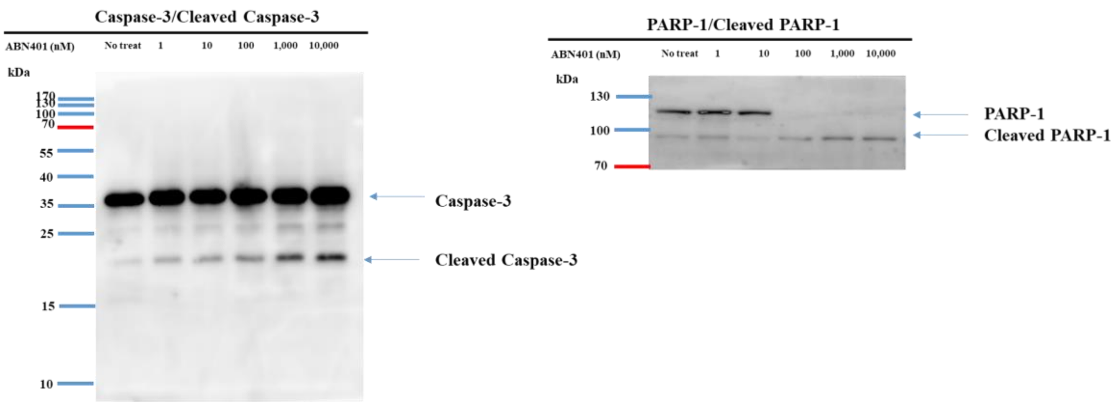

* Figure 2E – H1993

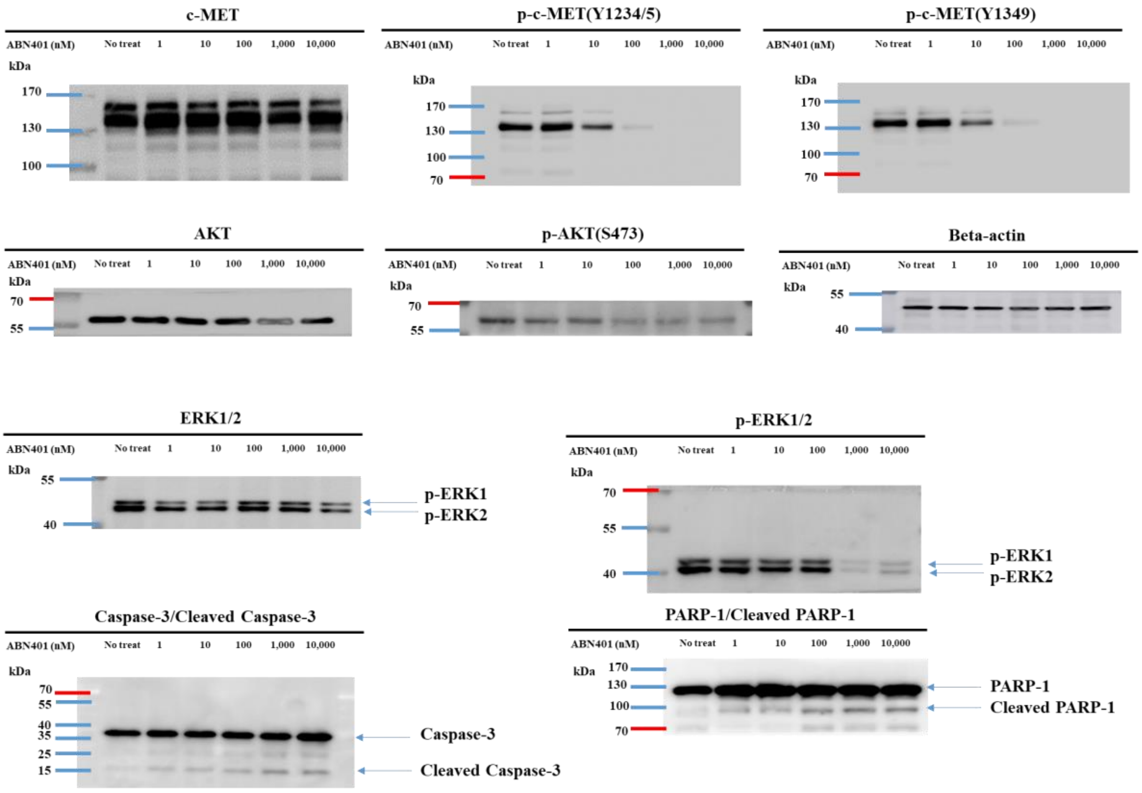

* Western blotting band (Intensity) – SNU5

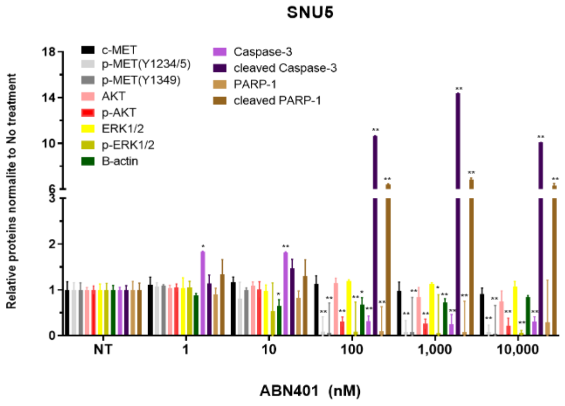

* Western blotting band (Intensity) – Hs746T

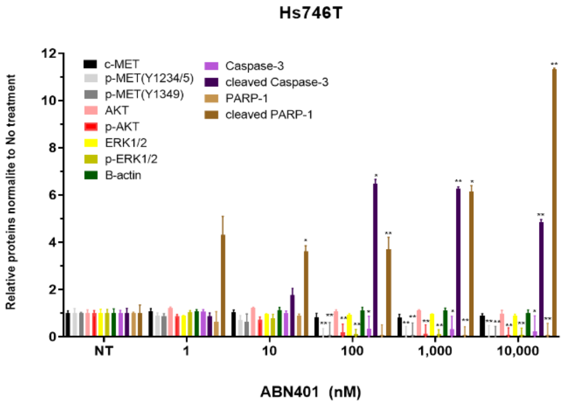

* Western blotting band (Intensity) – H1993

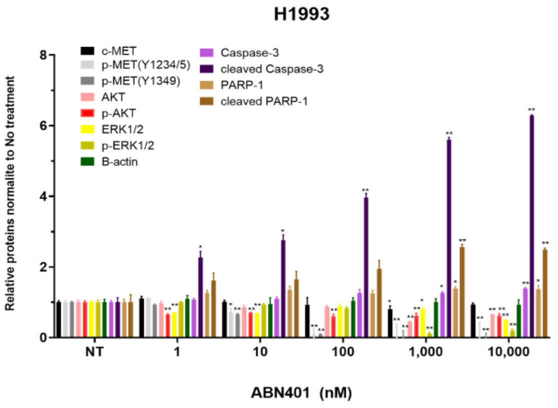

* Western blotting band (Intensity) – EBC-1

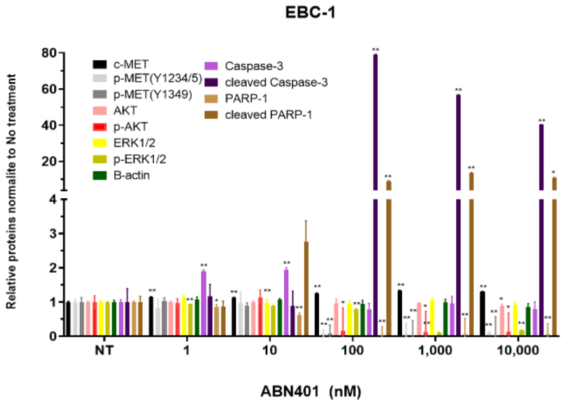

* Western blotting band (Intensity) – SNU638

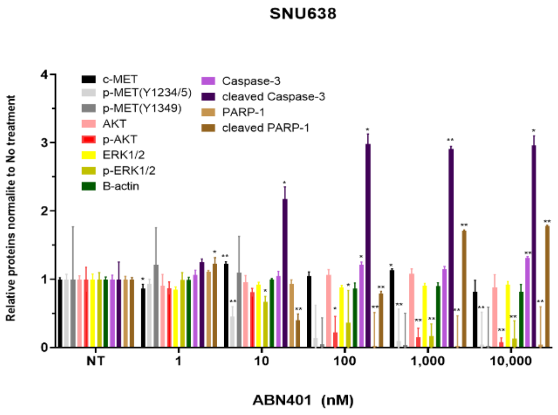

Figure S2. Uncropped Western blot bands for Figure 2. The level of total proteins or phosphorylated proteins or cleaved proteins were analyzed by western blotting. The uncropped Western blot bands related to Figure 2B~2E. The western blot bands were quantified using Image J. Data are represented as mean ± SEM. * $p < 0.05$, ** $p < 0.01$.

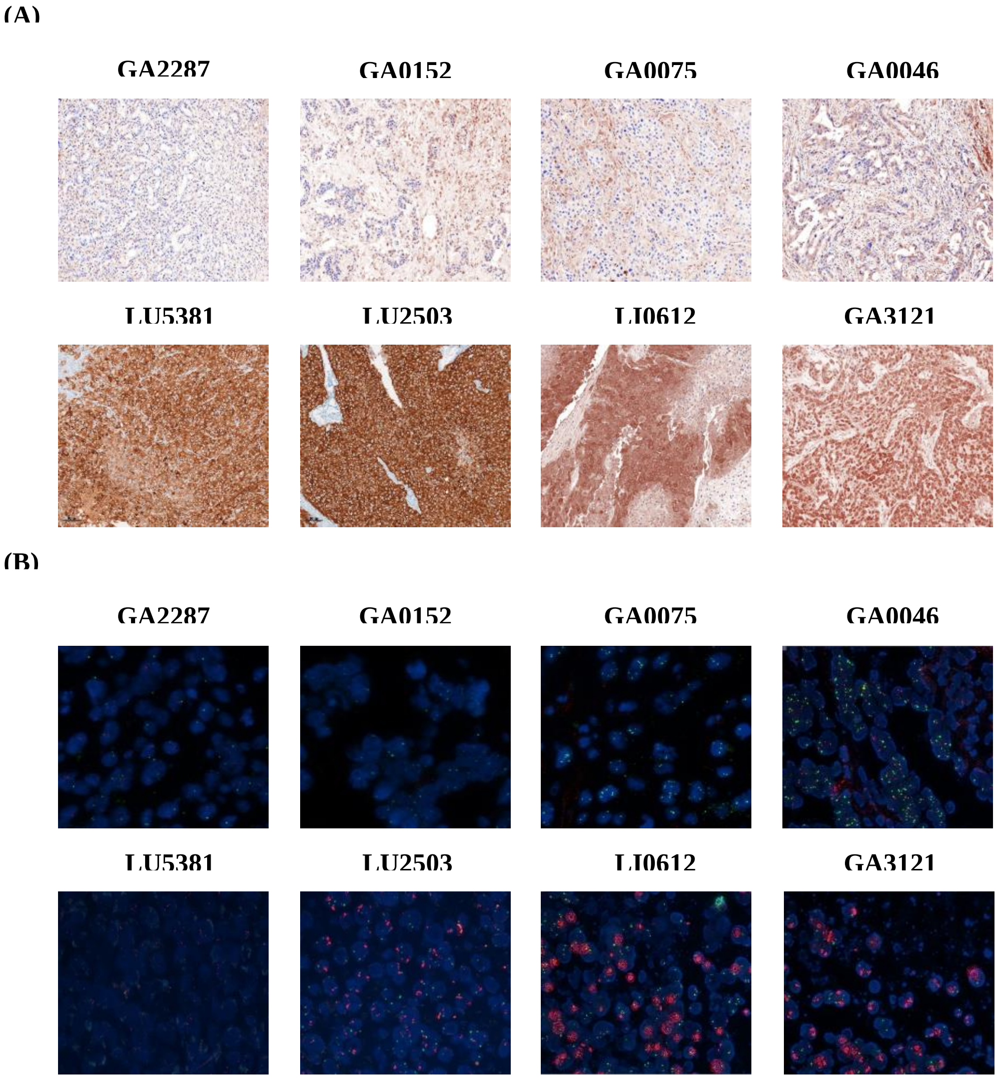

Figure S3. The MET status in patient-derived xenograft (PDX) models. (**A**,**B**) c-MET protein expression was measured by Immunohistochemistry (IHC), and Copy Number Variation (CNV) was analyzed by Fluorescence In situ hybridization (FISH) in patient-derived xenograft models with aberrant c-MET. LU5381, LU2503, LI0612 and GA3121 models had IHC intensity 3+ (A). In the FISH analysis, all Patient-derived xenograft (PDX) models with IHC intensity 3+ had a MET/CEP7 ratio > 5 except LU5381 (MET/CEP7 = 2.05) (B).
